# Correction

**DOI:** 10.1080/14756366.2024.2374156

**Published:** 2024-07-29

**Article title:** Discovery of novel benzohydroxamate-based histone deacetylase 6 (HDAC6) inhibitors with the ability to potentiate anti-PD-L1 immunotherapy in melanoma

**Authors:** Xiaopeng Penga, Ziwen Yua, Goverdhan Surinenia, Bulian Denga, Meizhu Zhanga, Chuan Lia, Zhiqiang Suna, Wanyi Pana, Yao Liu, Shenglan Liu, Bin Yu and Jianjun Chena

**Journal:** *Journal of Enzyme Inhibition and Medicinal Chemistry*

**Bibliometrics:** Volume 38, Number 01

**DOI:** https://doi.org/10.1080/14756366.2023.2201408

Unfortunately, during the post-processing of Figure 8A, an error occurred which led to the duplication of tumour tissues. The authors have now corrected the original figure and revised the corresponding description in the text, and all authors have agreed to the changes. The authors sincerely apologise for this oversight.

The correct figure should be:


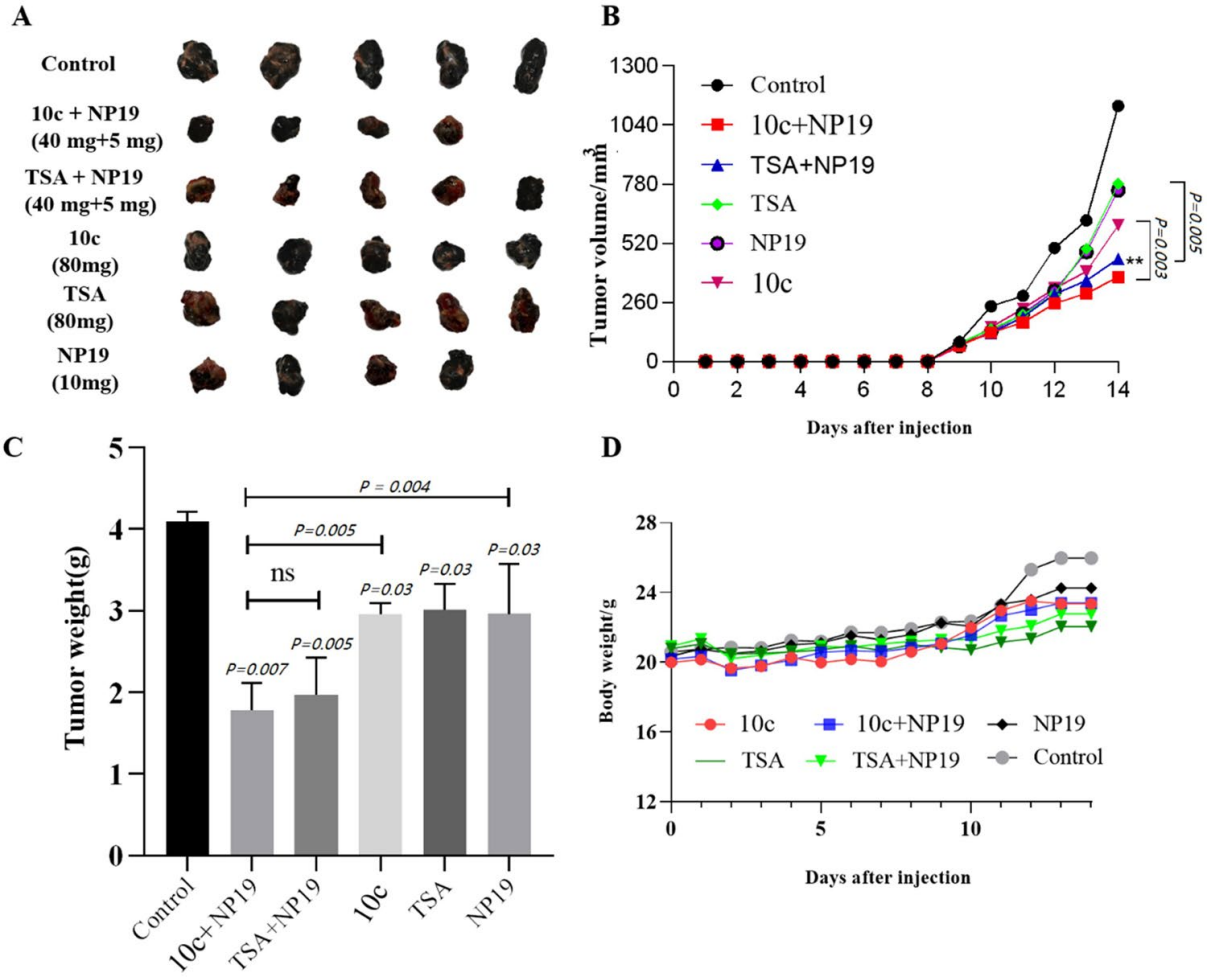


The correct statement under Experimental – In vivo antitumor efficacy study and safety profiling is “The in vivo antitumor efficacy of 10c was assessed following the protocol approved by National Institutional Animal Care and Ethical Committee at Southern Medical University. The 6–8 weeks old male C57 mice were subcutaneously injected with B16-F10 cells (4 * 10^6^/mL). The mice were divided into six treatment groups (six mice in each group). 10c and tubastatin A were dissolved in PBS: PEG300 (1:1). During the experiment, the tumour size and body weight were measured every day. Tumour growth inhibition was calculated by dividing the tumour volumes from treatment groups by those of the control groups as 100%. The harvested organs (liver, heart, and kidney) were processed into paraffin routinely, stained with haematoxylin and eosin (H&E) and further evaluated for the in vivo safety profiles of the test compounds”.

